# Contribution of the Australian field epidemiology training workforce to the COVID-19 response, 2020

**DOI:** 10.5365/wpsar.2022.13.4.979

**Published:** 2022-12-27

**Authors:** Amy Elizabeth Parry, Charlee Law, Davoud Pourmarzi, Florian Vogt, Emma Field, Samantha Colquhoun

**Affiliations:** a National Centre for Epidemiology and Population Health, College of Health and Medicine, Australian National University, Canberra, Australian Capital Territory, Australia.; b The Kirby Institute, University of New South Wales, Sydney, New South Wales, Australia.

The range of public health emergencies that occurred in Australia during 2020 illustrates the complexity of contemporary acute public health issues. In 2020 alone, Australia mounted responses to bush-fires, storms, drought, floods and rodent plagues, as well as the coronavirus disease (COVID-19) pandemic. Such events have highlighted not just the vital role played by the field epidemiology workforce in rapidly and effectively managing a wide range of public health emergencies but also the need to continually train and invest in this workforce to ensure high levels of public health emergency preparedness. (*1*-*5*)

Health workforce strengthening is essential to achieving the International Health Regulations (IHR 2005) core capacities. (*6*) The Asia Pacific Strategy for Emerging Diseases and Public Health Emergencies (APSED III) also makes specific reference to the need for a skilled and experienced local public health workforce for preventing the escalation of public health emergencies. (*7*)

The Australian Field Epidemiology Training Programme (FETP), commonly known as the Master of Philosophy in Applied Epidemiology (MAE), is one of several public health training programmes in Australia. Established in 1991 to address a recognized gap in the public health workforce, the programme is Australia’s accredited FETP. (*8*, *9*) Using the approach of “learning through doing,” students spend most of the 22-month-long programme working within a field placement. This approach ensures alumni are appropriately trained to contribute to the detection, investigation, response and control of acute public health events. (*8*) As of June 2021, there were 58 students and 255 alumni in the network.

Australian FETP alumni work in senior roles in health departments at local, state, national and international levels, in Aboriginal and Torres Strait Islander health services and organizations, in United Nations agencies, as well as in research institutions and academia. Alumni and students have been consistently involved in national and international epidemic responses, including severe acute respiratory syndrome (SARS) (2002–2003), H1N1 influenza (2009), Middle East respiratory syndrome coronavirus (MERS-CoV) (2012–) and Ebola virus disease in West Africa (2014–2016). The experiences of alumni and students have been used to modify the programme to make it more relevant, adaptive and “pandemic ready.”

The aim of this study was to describe the level and scope of Australian FETP alumni and student contributions to the COVID-19 response during the first 10 months of the pandemic so that these experiences could inform programme learning priorities going forward.

## Methods

In 2020, the Training Programs in Epidemiology and Public Health Interventions Network (TEPHINET) developed a survey to document the contribution of FETP trainees and alumni to COVID-19 preparedness and response internationally. (*10*) We adapted this instrument to conduct a cross-sectional survey of Australian FETP network members (survey available upon request to the corresponding author). Our survey collected participants’ demographic data and information about their employment and role in the COVID-19 response. Invitations to participate, with a link to the survey, were e-mailed to alumni and students in July 2020. Participants were also recruited using convenience and snowball sampling methods, with recipients asked to share the invitation with other Australian FETP alumni.

Roles were categorized into 10 main areas; each main area was assigned a list of associated activities. Multiple answers within each category were allowed. Open-ended questions were included to obtain additional details about participants’ roles and responsibilities. Data were stratified and descriptively analysed by category using Stata 15 (StataCorp LLC, College Station, TX, United States of America).

## Results

We received 66 responses, 57 from alumni (86%) and nine (14%) from current students. The majority (89%, 59/66) were involved in COVID-19 response activities in Australia; within this group, 61% (36/59) reported working for state or territory government departments, 10% (6/59) for a federal government department and 3% (2/59) for a local government department. Other workplaces included nongovernmental agencies (5%, 3/66), universities (21%, 14/66) and Aboriginal and Torres Strait Islander health services and organizations (3%, 2/66), with some respondents reporting multiple workplaces. Seven respondents reported working internationally (11%, 7/66).

Information on participant involvement in 43 COVID-19-related response activities is summarized in Table 1. Of the 66 respondents, 65 (98%) were involved in at least one listed activity and 36 (55%) in more than five activities. Over two fifths of respondents reported being involved in “surveillance” (82%, 54/66); 80% (53/66) were engaged in “reporting of data” and 71% (47/66) in activities related to “‘incident command.”

**Table 1:** Australian FETP survey respondents’ involvement in COVID-19 response activities, July–December 2020 (*n* = 66)

Category	Associated activities	n (% of total)
Surveillance	NOT involved in surveillance activities	12 (18%)
	Active disease surveillance	33 (50%)
	Case-based reporting	29 (44%)
	Contact tracing	26 (39%)
	Dissemination of case definitions	16 (24%)
	Other	33 (50%)
Reporting of data	NOT involved in reporting of data	13 (20%)
	Development of internal situation reports	41 (62%)
	Writing short reports or papers for publication in peer-reviewed journals	26 (39%)
	Sharing information on dashboards	18 (27%)
	Other	6 (9%)
Incident command	NOT involved in incident command activities	19 (29%)
	Emergency operations centre	22 (33%)
	Incident command system	16 (24%)
	Emergency management	9 (14%)
	Aboriginal and Torres Strait Islander health services and organizations	6 (9%)
	Other	12 (18%)
Operational research	NOT involved in operational research	21 (32%)
	Surveillance research	19 (29%)
	State, province, country-level coordination, regional or national planning and monitoring research	8 (12%)
	Risk assessment research	6 (9%)
	Community engagement research	2 (3%)
	Other	20 (30%)
Risk communication & community engagement	NOT involved in risk communication and community engagement activities	29 (44%)
	Development of communication for health-care providers	19 (29%)
	Media briefs and/or interviews	19 (29%)
	Construction of information sheets for the public	15 (23%)
	Construction of material for open access web pages for communication to the public	14 (22%)
	Communication for Aboriginal and Torres Strait Islander health workers or communities	11 (17%)
	On call for community queries	13 (20%)
	Hotline	5 (8%)
	Other	3 (5%)
Infection prevention and control (IPC)	NOT involved in IPC activities	42 (64%)
	Reporting and investigating cases of health-care-associated infections	11 (17%)
	Training staff in IPC	6 (9%)
	IPC risk assessment in facilities	6 (9%)
	Development of guidelines for IPC in facilities	5 (8%)
	Implementation of triage and control measures	2 (3%)
	Other IPC activities	5 (8%)
Operational support	NOT involved in operational support or logistics activities	45 (68%)
	Preparation of staff surge capacity and deployment mechanisms	15 (23%)
	Review of supply chain control and management system for medical and other essential supplies	2 (3%)
	Other	8 (12%)
Laboratory	NOT involved in laboratory activities	46 (70%)
	Standard operating procedures adopted for specimen collection and transportation for diagnostics	3 (5%)
	Access to designated COVID-19 reference laboratories	3 (5%)
	Development of surge plans to manage increased demand for testing	3 (5%)
	Conducting whole genome sequencing	2 (3%)
	Vaccine development for COVID-19	0 (0%)
	Development of rapid tests	0 (0%)
	Development or trial of point-of-care tests	0 (0%)
	Clinical trials for medications or vaccines	0 (0%)
	Other	11 (17%)
Point of entry	NOT involved in point-of-entry activities	48 (73%)
	Preparation of isolation facilities or quarantine measures	4 (6%)
	Communication of information about COVID-19 to travellers	2 (3%)
	Establishing standard operating procedures equipping staff to manage ill passengers	2 (3%)
	Other	12 (18%)
Case management	NOT involved in case management activities	52 (79%)
	Guidance made available for self-care of patients with mild symptoms	4 (6%)
	Health-care facilities prepared for high volume of cases	3 (5%)
	Dedicated teams to transport and treat suspected and confirmed cases	3 (5%)
	Other	6 (9%)

Respondents were allowed to give multiple answers within each main topic area. Percentages do not therefore sum to 100%.

Within the “surveillance” category, the most frequently reported activities included active disease surveillance (50%, 33/66), case-based reporting (44%, 29/66) and contact tracing (39%, 26/66) (**Table 1**). Other activities mentioned by participants included establishing customized COVID-19 surveillance systems, developing dashboards, responding to outbreaks on cruise ships and providing expert advice within a variety of settings.

Commonly reported activities in the “reporting of data” category included developing internal situation reports (62%, 41/66), preparing articles for publication in peer-reviewed journals (26/66, 39%) and sharing information on dashboards (27%, 18/66). Within the “incident command” category, the most frequently reported activity was involvement in emergency operations centres (33%, 22/66), followed by involvement in incident command systems (24%, 16/66). Six (9%) respondents were engaged in incident command activities within Aboriginal and Torres Strait Islander health services and organizations (**Table 1**).

Sixty-eight per cent (45/66) of participants reported involvement in “operational research” and 56% (37/66) participated in one or more activities related to “risk communication and community engagement.” Over a quarter (29%, 19/66) were involved in the preparation of communication for health-care providers; an equal number (29%, 19/66) interacted with or provided information to media (**Table 1**). All seven (11%) respondents working in international COVID-19 response reported participating in activities related to the development of risk communication briefings and messages.

Around a third of participants reported involvement in “infection prevention and control” (36%, 24/66), “operational support” (32%, 21/66) and “laboratory” (30%, 20/66) activities. Fewer respondents reported being involved in activities relating to “point of entry” (27%, 18/66) and “case management” (21%, 14/66) (**Table 1**).

## Discussion

Our survey revealed that Australian FETP alumni and students were involved in a wide range of pandemic response activities during the early months of the COVID-19 pandemic, suggesting that the programme provides a relevant and important contribution to the health response workforce in Australia and internationally. Alumni and students have been providing support during the COVID-19 pandemic in a variety of settings, including the public sector, academia and nongovernmental agencies, with many seconded into surge capacity roles.

The ability to redeploy the skilled field epidemiology workforce has been essential to the COVID-19 response at state, national and international levels. (*2*) Public health training programmes, such as the Australian FETP, need to remain responsive to workforce needs and continue to align with national, regional and international IHR workforce priorities. (*4*) That the programme is practical has been advantageous to the overarching Australian response by building a skilled and adaptive epidemiological workforce that is able to rapidly respond to acute public health emergencies.

Due to the sampling method used, it was not possible to accurately estimate the number of alumni the survey reached, with reasons for non-response remaining unknown. Therefore, the results presented are not generalizable to the Australian FETP population, though they do provide insight into some of the roles alumni and students played in the early phases of the response.

The Australian FETP has trained public health professionals who have contributed to different aspects of the COVID-19 response. The programme needs to continually adapt to ensure the training it provides remains relevant and addresses the breadth of skills required of field epidemiologists. It is important that support for the programme is maintained so that it can continue to play its critical role in building Australia’s public health capacity.
